# Downregulation of mitochondrial complex I induces ROS production in colorectal cancer subtypes that differently controls migration

**DOI:** 10.1186/s12967-023-04341-x

**Published:** 2023-08-03

**Authors:** Jean Bastin, Marine Sroussi, Ivan Nemazanyy, Pierre Laurent-Puig, Sophie Mouillet-Richard, Fatima Djouadi

**Affiliations:** 1 Centre de Recherche des Cordeliers, INSERM U1138, Sorbonne Université, Université Paris-Cité, 15, Rue de l’Ecole de Médecine, 75006 Paris, France; 2 Laboratoire de Biochimie, Ecole Supérieure de Physique et de Chimie Industrielle de la Ville de Paris, 75005 Paris, France; 3 Plate Plateforme d’étude du Métabolisme, SFR Necker, INSERM US24/CNRS UAR3633, 75015 Paris, France; 4 Institut du Cancer Paris CARPEM, Department of Biology Hôpital Georges Pompidou, 75015 Paris, France

**Keywords:** Colorectal cancer, Mitochondria, Complex I, Reactive oxygen species, SOD2, Consensus Molecular Subtypes (CMS), Epithelial-Mesenchymal Transition (EMT)

## Abstract

**Background:**

Colorectal cancer (CRC) can be classified into four molecular subtypes (CMS) among which CMS1 is associated with the best prognosis, while CMS4, the mesenchymal subtype, has the worst outcome. Although mitochondria are considered to be hubs of numerous signaling pathways, the study of mitochondrial metabolism has been neglected for many years. Mitochondrial Complex I (CI) plays a dual role, both in energy and reactive oxygen species (ROS) production. However, the possible contribution of CI to tumorigenesis in cancer remains unclear. The purpose of this study was to investigate the CI under the prism of the CMS classification of CRC in ex vivo models.

**Methods:**

Biochemical dosages, bioenergetics analysis and western-blot were used to characterize CI expression, function and redox balance in LoVo and MDST8 cell lines, belonging to CMS1 and CMS4 subgroups, respectively. Cell proliferation and migration were assessed by xCELLigence technology. Overproduction or scavenging of mitochondrial ROS (mtROS) were performed to analyze the effect of mtROS on proliferation, migration, and mesenchymal markers. Focal adhesion kinase (FAK) and its activation were analyzed by immunofluorescence. We assessed the distribution of two CI scores in CRC cohorts according to CMS classification and their relevance for patient survival.

**Results:**

We found that CI is downregulated in CMS4 cells and is associated with elevated mtROS. We establish for the first time that in these migrating cells, mtROS production is maintained at optimal levels not only through changes in CI activity but also by inactivation/acetylation of superoxide dismutase 2 (SOD2), a major mitochondrial antioxidant enzyme. We show that promoting or scavenging mtROS both mitigate CMS4 cells’ migration. Our results also point to a mtROS-mediated focal adhesion kinase (FAK) activation, which likely sustains their migratory phenotype. Using cohorts of CRC patients, we document that the expression of CI is downregulated in the CMS4 subgroup, and that low CI expression is associated with poor prognosis. Patients’ datasets reveal an inverse correlation between CI and the epithelial-mesenchymal transition (EMT) pathway.

**Conclusion:**

We showed that inhibition of CI contributes to heighten mtROS, which likely foster MDST8 migration and might account for the specific EMT signature of CMS4 tumors. These data reveal a novel role of mitochondrial CI in CRC, with biological consequences that may be targeted with anti- or pro-oxidant drugs in clinical practice.

**Supplementary Information:**

The online version contains supplementary material available at 10.1186/s12967-023-04341-x.

## Introduction

Colorectal cancer (CRC) is the third most common type of cancer in humans and the second leading cause of cancer death worldwide [1, 2]. In recent past years, the explosion of knowledge regarding molecular and biochemical alterations associated with the evolution of CRC has revealed that CRC is a much more heterogeneous disease than initially thought. This has led a consortium of expert groups to propose a consensus classification of CRC allowing to categorize most tumors into one of four consensus molecular subtypes (CMS), CMS1, CMS2, CMS3 and CMS4, based on transcriptional profiles, which are actually associated with distinct molecular and clinical features [3]. Among those subtypes, CMS1, which is characterized by microsatellite instability and high immune infiltration, has a much better prognosis than the CMS4 subtype, which has a particularly dismal prognosis, likely due to specific hallmarks of CMS4 tumors such as a prominent epithelial-mesenchymal transition (EMT) signature and the activation of transforming growth factor (TGF)-β signaling. Although the CMS classification represents a major advance in the understanding of CRC heterogeneity, many fundamental aspects remain to be explored. In particular, still little is known on the metabolic pathways involved in the specificities of the various CMS subtypes. Indeed, ascribing a specific metabolic signature to each CMS subgroup could help refine CRC diagnosis, identify new therapeutic targets, and/or predict response to treatment.

Metabolic reprogramming was recognized as a hallmark of cancer in 2011 [4]. Most scientists in this field have focused their attention on glycolysis, or on its metabolic intermediates that provide building blocks for the synthesis of amino acids, fatty acids and nucleotides during oncogenesis and tumor progression [5]. Thus, for several decades, the Warburg effect, i.e. the fact that cancer cells preferentially metabolize glucose via aerobic glycolysis, has occulted the contribution of mitochondria to many features of tumor cells to support their malignancy beyond the obvious energy supply [6]. Moreover, the predominance of Warburg’s theory has led to the general misconception that cancer cells are exclusively glycolytic, and, that the “switch off” of mitochondrial functions is one of the major cellular features accounting for cancer initiation and progression [7].

In the mitochondria, complex I (CI) has a peculiar and very important role in the respiratory chain (RC), as the gatekeeper of RC and as the node point in NADH metabolism, initiating the electron transfer from NADH to the other respiratory complexes to generate ATP [8]. In recent years, an increasing number of studies have investigated a possible role of CI in various aspects of cancer biology and have revealed that CI dysfunction in different cancers can be associated to tumor progression and metastasis or, to the opposite, inhibition of tumor growth, highlighting the oncojanus face of CI [9, 10]. Nevertheless, in most of these reports, the biochemical and functional consequences of these CI alterations were not studied and thus remain to be elucidated. Importantly, CI is, with Complex III, the main site of ROS production in the mitochondria [8]. Although, it is widely acknowledged that ROS generation participates to many steps of metastasis [11], the contribution of mitochondrial ROS to these steps, in particular EMT, is still understudied.

Here, we sought to investigate the mitochondrial CI under the prism of the CMS classification of colorectal cancer using two CRC cell lines: LoVo and MDST8, belonging to the CMS1 and CMS4 subgroups, respectively. We find that CI activity is lower in MDST8 vs LoVo cells and that this decrease, which generates mitochondrial ROS (mtROS), contributes to the migratory potential of MDST8 cells. We find that the down-regulation of CI is associated to an acetylated/inactivated SOD2 protein in MDST8, which likely participates to heighten mtROS. Furthermore, we provide evidence that specific overproduction or scavenging of mtROS promotes different effects on proliferation and migration in the two cell lines. We also highlight a putative activation of focal adhesion kinase by mtROS that may foster MDST8 cell migration. Finally*, *in silico analysis of patient datasets indicates that the CI decrease has relevance with respect to CRC heterogeneity and prognosis.

## Methods

### Cell culture

The human CRC LoVo and MDST8 cell lines were purchased from the European Collection of Authenticated Cell Cultures. Cell lines were cultured in DMEM with 10% fetal bovine serum and 0.2% primocin (InvivoGen), at 37 °C, 5% CO_2_. For treatments, cells were incubated in fresh media containing 0.1 µM Rotenone or 50 µM MitoTempo or vehicle (0.01% DMSO). Unless otherwise mentioned, the cells were treated for 48 h.

### Cell proliferation

Cells (2 × 10^5^) were seeded in several 6-well plates and incubated for 24 h, 48 h or 72 h. After incubation, total cell numbers from three different wells were determined using a CASY TT cell counter (Schärfe System GmbH, Reutlingen, Germany).

### Electric impedance measurement

Cells’ proliferation/adhesion and migration were examined using the xCELLigence Real-Time Cellular Analysis (RTCA) system (ACEA Biosciences, Inc), that measures in real-time electrical impedance, which reflects the number of cells, but also cell size and morphology, and cell attachment quality. The impedance is reported using a parameter termed cell index. Cells were grown with or without treatment 48 h prior seeding. For studies of proliferation/adhesion, cells were trypsinized and seeded at 10,000 cells per well in a volume of 0.2 ml on 16-well plates (E-plates) with microelectrodes on the bottom of each well. For migration studies, CIM plates (16 wells) were used. In these plates, the cells were seeded (30,000 cells per well in a volume of 0.1 ml) on the upper compartment of the system in a serum-free medium and allowed to migrate to the lower compartment containing a medium with 10% FBS used as a chemoattractant. The impedance was monitored every 10 or 15 min for 48 or 96 h at 37 °C, 5% CO_2_ and expressed as cell index (CI). In each experiment, triplicates or quadruples of each condition were run. The data were analyzed using the RTCA software and were normalized to the impedance value of each well at the beginning of the experiments.

### Complex I enzyme activity

Mitochondria were isolated from cell lines using a mitochondria isolation kit for cultured cells (Abcam) according to the manufacturer’s instructions. The method used for the measurement of respiratory chain CI activity was adapted from the method developed by Janssen et al. [12]. CI enzyme activity was assayed spectrophotometrically in 96-well plates based on the kinetics of decrease of dichloroindophenol (DCIP) absorption at 614 nm. The reaction medium (250 µl/well, 37 °C) contained 25 mM KH2PO4/K2HPO4 pH 7.8, 3.5 mg/ml BSA, 70 µM DCIP, and 100 µM decylubiquinone. After addition of mitochondria (5 µl/well) and reading of basal absorbance for 1 min, the reaction was initiated by addition of 200 µM NADH. After monitoring the decrease in DCIP absorbance for 2 min, 1 µM rotenone was then added in each well and the readings were continued for 2 min. The CI enzyme activity was defined as the rotenone sensitive fraction i.e. was calculated from the difference in absorption slopes before and after addition or rotenone. CI activity was expressed as nanomoles of DCIP oxidized per minute per mg of protein.

### Oxygen consumption rate (OCR)

The experiments were performed by the platform for metabolic studies at SFR Necker. Briefly, cells were plated in cell culture XF96 microplates (Agilent technologies) at 2 × 10^4^ cells/well and the cellular OCR was determined by a Seahorse Bioscience XF96e extracellular flux analyzer (Agilent technologies) according to the manufacturer’s instructions. Briefly, cells were balanced for 1 h in XF assay media (Agilent Technologies) supplemented with 2 mM Glutamine, 10 mM Glucose and 1 mM Sodium Pyruvate. Compounds were injected during the assay at the following final concentrations: Oligomycin (ATP synthase inhibitor, 1 μM), FCCP (uncoupling agent measuring the maximal respiration capacity; 1 μM), Rotenone and Antimycin A (ETC inhibitors; 1 μM). For each cell line 6–12 technical replicates were evaluated. All OCR measurements were normalized to the protein concentration dosed at the end of every experiment.

### Western blot analysis

Cells were harvested in RIPA buffer containing: 50 mM Tris–HCl pH 8, 150 mM NaCl, 0,5% Nonidet P40, 0,25% sodium deoxycholate, 0,1% sodium dodecyl sulfate (SDS), 1 mM phenylmethylsulfonyl fluoride, 1 × protease inhibitor cocktail, (Complete mini, Roche), 1 × Phosphatase inhibitor cocktail (PhosSTOP, Roche), 10 mM Nicotinamide, to prepare total protein extracts. Protein concentration was determined by the Lowry method. Protein extracts (15-20 µg) were run in BoltTM 10% Bis–Tris Plus gels (Invitrogen) and transferred to PVDF membranes (Biorad). Membranes were blocked with 5% milk or 5% BSA in 1 × TBS-T for 1 h before incubation with primary antibodies overnight at 4 °C. Immunoreactive bands were analyzed with a LAS-4000 luminescent image analyzer. The results were expressed as arbitrary units normalized to the amount of α-tubulin. The following antibodies were used: NDUFV1 (Proteintech, ref: 11238-1-AP), NDUFS1 (Abcam, ref: ab169540), NDUFS3 (Abcam, ref: ab110246), SOD2 (Abcam, ref: ab68155), acetylK68-SOD2 (Abcam, ref: ab137037) N-cadherin (BD Biosciences, ref: 610920), ZEB1 (Abcam ref: 203829), tubulin (Sigma-Aldrich, ref: T9026), p-FAK-Y^397^ (Abcam, ref:ab81298), FAK (Cell Signaling, ref: 3285).

### Total RNA isolation and RT-PCR analysis

RNAs were isolated using the RNeasy extraction kit (Qiagen, Limburg, Netherlands), as recommended by the manufacturer's instructions. cDNAs were generated from 1 µg of total RNA using the High-capacity cDNA Reverse Transcription (Applied Biosystems) and quantified in triplicates on the 7900HT Fast Real-Time PCR system (Applied Biosystems) using Absolute qPCR SYBR Green ROX Mix (Thermo Scientific). The primer sequences are shown below: ZEB1-F: AAGAATTCACAGTGGAGAGAAGCCA; ZEB1-R: CGTTTCTTGCAGTTTGGGCATT; CDH2-F: ACAGTGGCCACCTACAAAGG; CDH2-R: ACAGTGGCCACCTACAAAGG; RPL13A-F: CCTGGAGGAGAAGAGGAAAGAGA; RPL13A-R: GAGGACCTCTGTGTATTTGTCAA. The results are expressed as the relative quantification of a target gene transcript normalized to RPL13A housekeeping gene, using the ΔΔCt method.

### Gene expression

The following datasets were retrieved from public sources GSE39582 (“CIT cohort”, n = 566), GSE14333, GSE17536, GSE17537, GSE33113, altogether forming the “validation cohort” (n = 431); subtype classification systems assignments were performed using original published predictor methods as described in [3].

### Gene set enrichment analysis (GSEA)

GSEA [13] was performed on the following data sets: GSE39582 [14] for patients and GSE59857 [15] for cell lines. The GSEA was performed using the Broad Institute platform (http://www.broadinstitute.org/gsea/index.jsp; Version 2.0.14).

### Mitochondrial ROS

Mitochondrial ROS were measured using Mitosox probe (Invitrogen). Experiments were performed in 24-wells in which cells were incubated with 1 µM Mitosox in PBS for 10 min at 37 °C. Cells were subsequently washed with PBS, lysed in 250 µl of 1 M NaOH and transferred to a 96-well black plate for measurement of fluorescence intensity with a plate reader (infinite^®^M200, Tecan). The results were normalized to the amount of protein in each well.

### Superoxide dismutase 2 enzyme activity

Determination of superoxide dismutase (SOD) enzyme activity in CRC cell lines was performed according to the spectrophotometric method of Paoletti et al., with minor modifications [16].

### Immunofluorescence

Briefly, cells grown on glass coverslips were washed and fixed with 4% formaldehyde in PBS. After permeabilization, cells were incubated with primary antibody diluted (1/250) in PBS enriched with 1% BSA and 0.1% Tween for 1 h at room temperature. The following antibodies were used: FAK (Abcam), p-FAK-Y^397^ (Abcam). F-actin fibers were stained with TRITC-Phalloidin (Sigma-Aldrich). Cells were then incubated with Alexa Fluor 488 secondary antibodies (1/1000) (Molecular Probes, Eugene, OR, USA) and DAPI, used as nuclear marker. Immunolabelling was observed and images recorded using a Zeiss Axio Observer Z1 at X40 magnification.

### Statistical analysis

The results are reported as the means ± SEM. All statistical analyses were performed using GraphPad Prism software (version 9.4.1). Data distribution was first checked for normality using the Shapiro–Wilk test, and parametric or non-parametric tests were then applied accordingly. Differences between groups were analyzed by Mann–Whitney test or paired or unpaired two-tailed Student’s t test for the comparison of two groups, or by Two-way ANOVA and the Tuckey test for comparison of four groups. Results from RNA analysis in cohorts are expressed as median and interquartile range. Statistical analysis was performed in R studio (version 2022.07.2) using one-way ANOVA followed by Wilcoxon rank-sum tests with Holm’s correction for multiple comparisons. Survival curves were obtained using Kaplan–Meier estimates using the Survival R package (version 3.5-0) and differences between groups of patients were assessed using the log-rank test for univariate analyses or Cox models for multivariate analyses. A p < 0.05 was considered significant.

## Results

### Mitochondrial Complex I is downregulated in the MDST8 cell line, a prototypical model of the CMS4 subtype

We focused on CMS1 and CMS4 CRC, which both arise from serrated precursor lesions (Reviewed in [17], yet are associated with opposite outcomes. We selected the LoVo and MDST8 cell lines as prototypical CMS1 (“good prognosis”) and CMS4 (“poor prognosis”) models, respectively [18, 19], and verified that the two cell lines exhibit distinct cellular behaviors. We first documented through cell counting that MDST8 cells proliferate around twice faster than LoVo cells (Additional file 1: Figure S1a). We also employed the xCELLigence real-time analysis system to monitor proliferation/adhesion (E-plates) as well as migration (CIM-plates). When cells were seeded on E-plates and allowed to proliferate for 48 h, the cell index for MDST8 increased much more rapidly than that of LoVo cells (Additional file 1: Figure S1b and c). Seeding on CIM-plates further revealed that MDST8 cells have the capacity to migrate while LoVo cells do not (Additional file 1: Figure S1d and e). Accordingly, western-blot analysis showed abundant expression of several EMT markers (N-cadherin, vimentin and ZEB1) in MDST8 cells, which were not detected in LoVo cells (Additional file 1: Figure S1f). Altogether, the specific cellular and molecular features of each cell line confirm that LoVo and MDST8 cells represent adequate models for CMS1 and CMS4 subtypes.

**Fig. 1:**
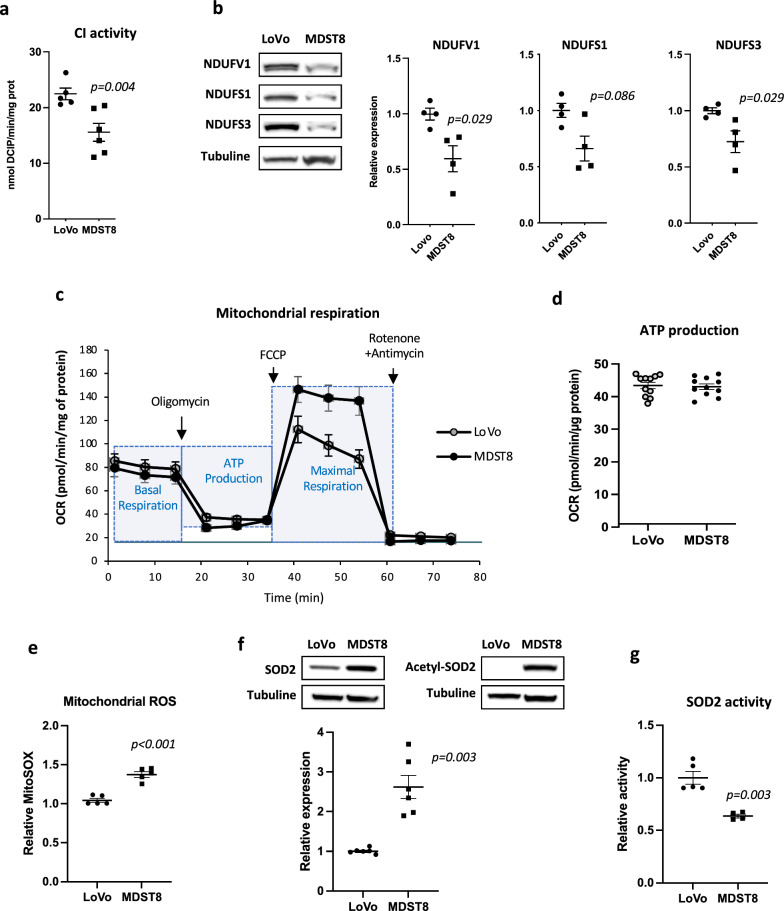
Downregulation of CI in MDST8 cells increases mtROS and is associated with acetylation of SOD2. **a** Complex I enzyme activity measurements (n = 6). **b** Representative immunoblot and quantification of three CI subunits’ protein levels by western-blot (n = 4). **c** and **d** Measurement of the oxygen consumption rate (OCR) using Seahorse Bioanalyser; representative experiment, n = 3 independent experiments were performed. For ATP production, the points represent technical replicates. **e** Measurements of mitochondrial ROS with Mitosox probe (n = 5). **f** Representative immunoblot and quantification of SOD2 and acetyl K68-SOD2 protein levels (n = 5–6). **g** Measurements of SOD2 enzyme activity (n = 5). Except for Seahorse experiments, the results are means ± SEM of n independent experiments. In certain experiments, samples were run in duplicates or triplicates. The p values are indicated in the figure

Having validated the two models, we measured CI enzyme activity in mitochondria-enriched homogenates of LoVo and MDST8 cells and found reduced (-31%) enzyme activity in MDST8 cells compared to LoVo cells (15.5 ± 1.6 and 22.5 ± 1.0 nmol DCIP/min/mg protein, respectively, p = 0.0043) (Fig. 1a). Complex I, the largest complex of respiratory chain, consists of 45 different subunits, 38 of which are encoded by nuclear genes that assemble to form three functional modules (N Module, Q Module and P Module) ensuring different functions. Seven of the nuclear-encoded subunits, NDUFV1, NDUFV2, NDUFS1, NDUFS2, NDUFS3, NDUFS7, NDUFS8 form the “catalytic core” since it binds and oxidizes NADH and is responsible for the electron transfer to the final acceptor ubiquinone [8]. We assessed the relative expression of three of the seven subunits constituting the CI catalytic core and showed that the amounts of NDUFV1, NDUFS1 and NDUFS3 proteins were significantly reduced in MDST8 cells, compared to LoVo cells (Fig. 1b). Since CI proteins and enzyme activity are reduced in MDST8 cells, we asked whether these changes translate into a decrease in mitochondrial respiration, as it can be surmised. Thus, using the Seahorse extracellular flux analyzer, we analyzed the oxygen consumption rate (OCR) in the presence of major cell energy substrates (glucose, pyruvate, glutamine), which provides a measure of mitochondrial respiration. Using mitochondrial inhibitors, the system allows the measurements of baseline OCR and calculation of corresponding mitochondrial ATP production and determination of maximal uncoupled respiration. As shown in Fig. 1c and d, Lovo and MDST8 cells exhibited similar basal OCR and ATP production values. OCR values in the presence of FCCP were higher in MDST8 compared to LoVo, reflecting higher respiratory chain capacities in MDST8 when mitochondrial oxygen consumption was uncoupled from ATP production.

### MDST8 cells exhibit a higher level of mitochondrial ROS than LoVo cells

Since the moderate but significant decrease of CI in MDST8 cells does not affect ATP production, we sought to determine whether this dysfunction could have other non-energetic biochemical consequences such as increased ROS production. Indeed, we and other have demonstrated that in fibroblasts from patients with inherited CI deficiency, superoxide production is increased [20, 21]. Here, we observed that mitochondrial ROS (mtROS) levels were significantly increased (+ 40%, p < 0.001) in MDST8 cells as compared to LoVo cells (Fig. 1e). Importantly, ROS levels are the result of a fine balance between ROS production and ROS scavenging. In mitochondria, the manganese superoxide dismutase (SOD2) is a first-line antioxidant defense enzyme, which protects cells from oxidative stress generated in these organelles. As shown in Fig. 1f, SOD2 protein level is twice higher in MDST8 cells compared to LoVo cells, while, surprisingly, the enzyme activity of SOD2 is significantly lower (Fig. 1g). It is well acknowledged that SOD2 is submitted to important post-translational modifications that finely regulate its activity [22]. Of crucial importance here, we previously showed that, in CI-deficient-cell, activation of SOD2 is directly linked of its deacetylation by Sirtuin-3 (SIRT3), a major mitochondrial NAD^+^ dependent protein deacetylase [20]. We found that SOD2 is highly acetylated in MDST8 cells (Fig. 1f) in coherence with the decreased SOD2 enzyme activity. Therefore, we may surmise that the higher level of mtROS measured in MDST8 cells is due both to a decrease in CI activity and to a decrease in SOD2 activity. These data point to different mtROS production and elimination in MDST8 versus LoVo cells.

### Boosting or scavenging mtROS levels both inhibit MDST8 cell migration

**Fig. 2:**
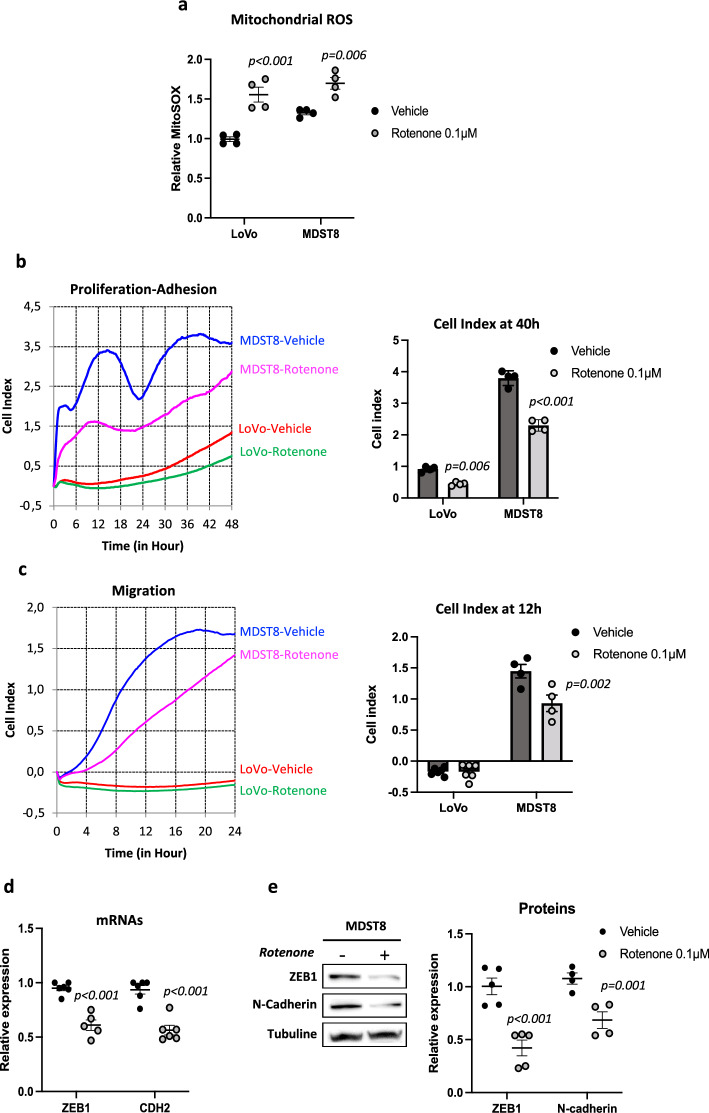
Rotenone enhances mtROS and decreases MDST8 cell migration. **a** Measurements of mitochondrial ROS with Mitosox probe. Cells were treated 48 h with 0.1 µM rotenone or vehicle (DMSO) (n = 4). **b** Representative kinetics of LoVo and MDST8 proliferation/adhesion using xCELLigence methodology and quantification of cell index at 40 h. Cells were treated 48 h prior seeding (n = 4). **c** Representative kinetics of cell migration using xCELLigence and quantification of cell index at 12 h (n = 4). **d** qRT-PCR analysis of ZEB1 and CDH2 in MDST8 cells (n = 3). **e** Representative immunoblot and quantification of ZEB1 and N-cadherin protein levels by western-blot analysis (n = 5). The results are means ± SEM of n independent experiments. In certain experiments, samples were run in duplicates or quadruplates. The p values are indicated in the figure

Having shown that LoVo and MDST8 cells exhibit differences in CI activity, ROS levels and cellular behavior (Additional file 1: Figure S1), we sought to assess a potential link between CI dysfunction and ROS production on the one hand and cellular features on the other hand. To this aim, we submitted cells to a mild inhibition of CI using rotenone, a usual inhibitor, at a concentration of 0.1 µM, which induces a partial but not total inhibition of CI activity [23]. As shown in Fig. 2a, rotenone significantly enhanced mitochondrial ROS production in both LoVo and MDST8 cells. We then assessed the functional consequences of this exposure to additional ROS. Both cell lines were treated with 0.1 µM rotenone 48 h prior plating in specific xCELLIgence plates, and allowed to proliferate or migrate. Rotenone significantly reduced proliferation/adhesion of both LoVo and MDST8 cells, as illustrated by Fig. 2b and Additional file 1: Figure S2. Of note, rotenone also significantly reduced the migratory phenotype of MDST8 cells, as shown by both cell index curves and cell index quantifications (Fig. 2c). We further monitored a down-regulation of ZEB1 and N-Cadherin markers in MDST8 cells after 48 h of rotenone treatment both at the mRNA and protein levels (Fig. 2d and e), which may account for the decreased migratory potential of these cells.

**Fig. 3:**
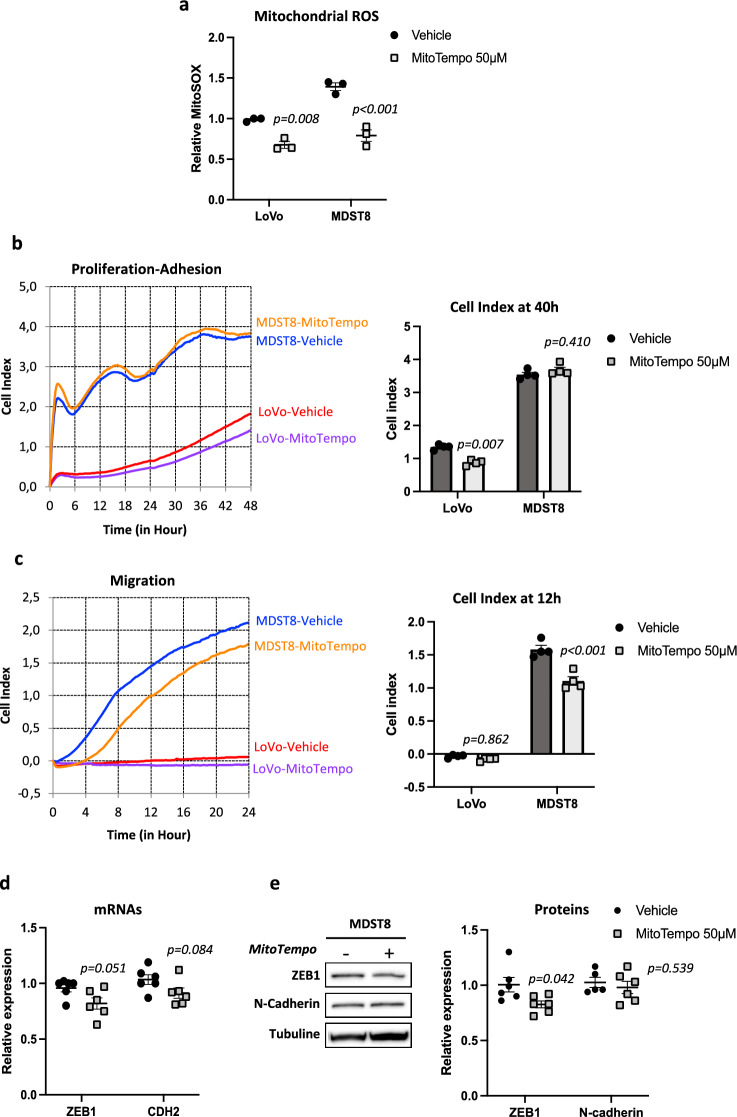
Mitotempo decreases mtROS and decreases MDST8 cell migration. **a** Measurements of mitochondrial ROS with Mitosox probe. Cells were treated 48 h with 50 µM MitoTempo or vehicle (DMSO) (n = 3). **b** Representative kinetics of LoVo and MDST8 proliferation/adhesion using xCELLigence methodology and quantification of cell index at 40 h. Cells were treated 48 h prior seeding (n = 4). **c** Representative kinetics of cell migration using xCELLigence and quantification of cell index at 12 h (n = 4). **d** qRT-PCR analysis of ZEB1 and CDH2 in MDST8 cells (n = 3). **e** Representative immunoblots and quantification of ZEB1 and N-cadherin protein levels by western-blot (n = 5). The results are means ± SEM of n independent experiments. In certain experiments, samples were run in duplicates or quadruplates. The p values are indicated in the figure

Next, in an opposite approach, we used MitoTempo, a specific scavenger of mitochondrial superoxide to probe the effects of mitochondrial ROS reduction. MitoTempo significantly lowered mitochondrial superoxide in LoVo and MDST8 by 27% and 40%, respectively (Fig. 3a). Analysis of xCELLigence profiles and cell index quantifications indicated that scavenging of mtROS decreased proliferation/adhesion in LoVo cell line (p = 0.007), while having no significant effect in MDST8 (Fig. 3b). Surprisingly, MDST8 cells pretreated 48 h with MitoTempo exhibited reduced migration (Fig. 3c) similar to the effects observed with rotenone. However, the effects of MitoTempo on EMT markers were less obvious than those of rotenone, with only slight decreases (Fig. 3d and e). Altogether, our results indicate that promoting mtROS production or scavenging mtROS both mitigate MDST8 cell migration but have no effect on LoVo cell migration.

### FAK activation contributes to MDST8 cells migration

**Fig. 4:**
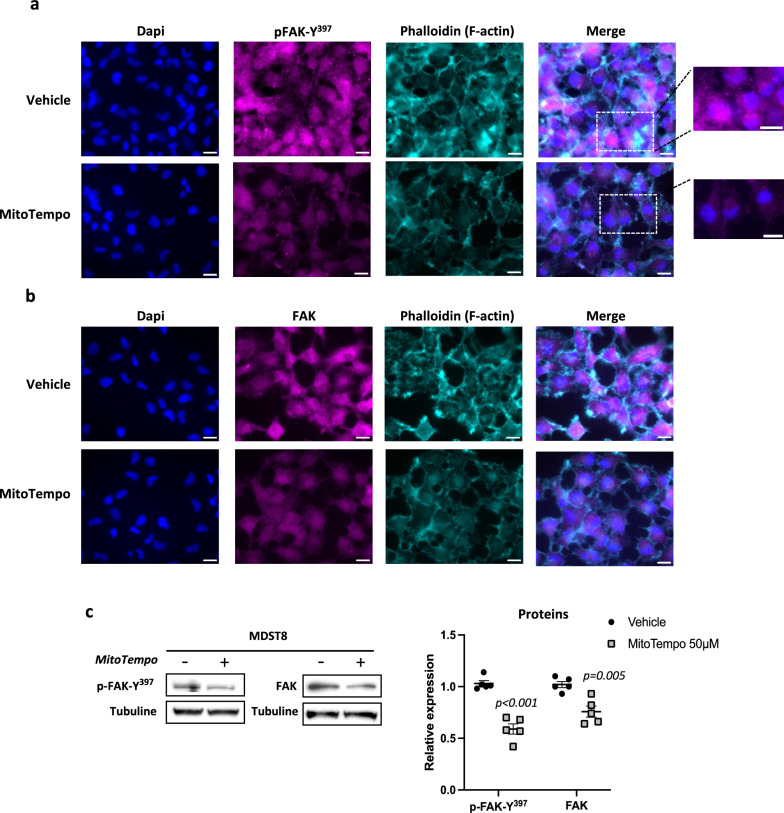
FAK activation upon oxidative stress likely contributes to MDST8 migration. **a** and **b** Representative images of immunofluorescence staining of pFAK-Y^397^ and FAK in vehicle and MitoTempo-treated (50 µM, 48 h) MDST8 cells. Dapi (blue), pFAK-Y^397^ and FAK (magenta), Phalloidin (cyan). Scale bar: 20 µm. **c** Representative immunoblots and quantification of pFAK-Y^397^ and FAK protein levels by western-blot (n = 5). The results are means ± SEM. The p values are indicated in the figure

Next, we went on to investigate a possible involvement of the focal adhesion kinase (FAK) in the effects of MitoTempo on MDST8 migration based on two main findings. First, FAK is a well-characterized tyrosine kinase known to play a central role in integrin-mediated signaling cascade at structures called focal adhesions (FAs), the formation and turnover of which are regulated dynamically during cell migration [24]. Second, ROS have been shown to influence cytoskeleton dynamics controlling cell motility and adhesion [25], and to modify the distribution of FAK within the cell [26]. The first step of FAK activation requires its autophosphorylation at Tyr397 (p-FAK-Y^397)^, which subsequently leads to phosphorylation of other sites [27]. We thus performed immunofluorescence analysis of p-FAK-Y^397^ and total FAK in MSDT8 cells. As shown in Fig. 4a and b, strong p-FAK-Y^397^ and FAK staining were observed in MDST8 cells, which were markedly decreased in MitoTempo-treated cells. The results were corroborated by western-blots analysis of p-FAK-Y^397^ and FAK protein levels (Fig. 4c). Interestingly, the immunofluorescence images also revealed an intense nuclear staining of p-FAK-Y^397^ in MDST8 cells that was lowered by MitoTempo (Fig. 4a), and FAK has been reported to translocate into the nucleus upon stress signals or cells’ detachment from the matrix [26, 28]. Finally, we observed a strong impact of MitoTempo treatment on the actin network of MDST8 cells (Fig. 4a and b). As a whole, these results indicate a ROS-dependent FAK activation in MDST8 cells, which likely sustains their migratory phenotype.

### CI expression is reduced in mesenchymal colorectal tumors and low CI levels are associated with poor prognosis

**Fig. 5:**
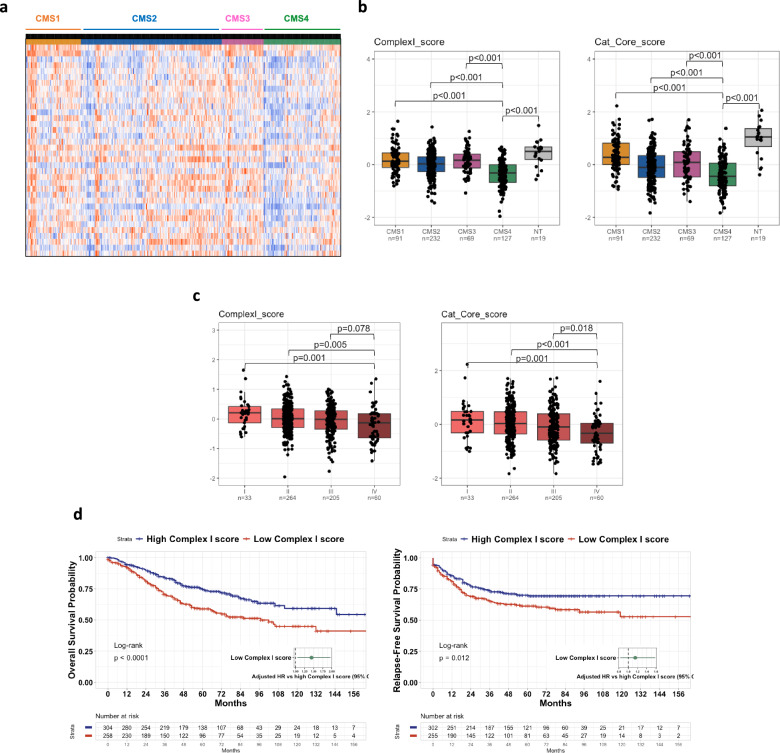
Complex I score is diminished in CMS4 subtype of CRC and is associated with poor prognosis. **a** Heatmap of the distribution of 38 genes (nuclear-encoded) of CI in colon cancer patients from the CIT cohort (n = 566) according to CMS classification. **b** Distribution of the Complex I score (mean of the 38 nuclear-encoded genes’ expression) and the Cat Core score (mean of the expression of the 7 subunits constituting the catalytic core of CI) in the 4 molecular subgroups of the consensus classification (CIT cohort). NT: non-tumors controls. **c** Distribution of the Complex I score and the Cat Core score in the CIT cohort according to the stages I to IV of CRC. **d** Kaplan–Meier overall survival (OS, left panel) and relapse-free survival (RFS, right panel) according to high and low complex I score were determined in the CIT cohort. Hazard ratios were adjusted for TNM stage, MMR status and adjuvant chemotherapy

**Fig. 6:**
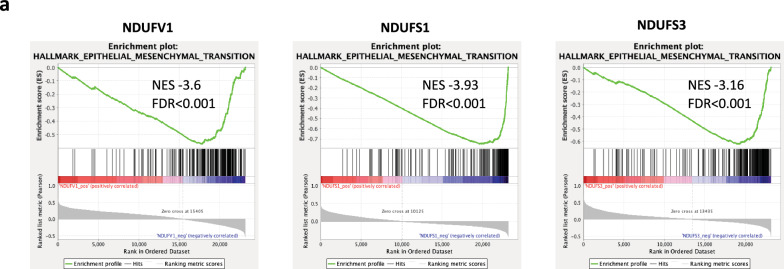
Three CI subunits’ transcripts are inversely correlated to EMT pathway. GSEA analysis showing the downregulation of the epithelial-mesenchymal-transition (EMT) signature in the genes most correlated with NDUFV1, NDUFS1 and NDUFS3 expression in the CIT cohort (n = 566). NES: normalized enrichment score

In a last step, we sought to assess the translational relevance of our findings through in silico analyses of patient datasets. We first leveraged the CIT cohort composed of n = 566 patients with stage I to IV CRC. The heatmap displayed in Fig. 5a represents the distribution of the 38 nuclear-encoded genes constituting the CI according to the CMS classification, and reveals a global under-expression of CI transcripts in CMS4 tumors versus other subgroups. Then, for a better understanding and quantification of these results, we decided to use two types of scores to assess the CMS-dependent distribution of CI transcripts: the “Complex I score” corresponding to the mean of the expression of the 38 nuclear-encoded genes, and the “Cat Core score” corresponding to the mean of the expression of the 7 subunits constituting the catalytic core of CI. When assessing the distribution of “Complex I score” according to the CMS classification, we found that the CI score was significantly decreased in CMS4 as compared to the other subtypes (Fig. 5b). It was also decreased as compared with non-tumor (NT) samples, which was also true for CMS2 (but not CMS1 nor CMS3) tumors. Next, when examining the catalytic core score, we found that it was significantly decreased in all CMS subgroups as compared with NT samples and had the lowest level in CMS4 among tumors. The expression of the seven catalytic core subunits was also assessed individually according to the CMS classification and the results confirmed their global decreased expression in CMS4 samples (Additional file 1: Figure S3). The downregulations of “Complex I score” and “Catalytic Core score” in CMS4 tumors compared with other CMS subtypes were confirmed in the validation cohort (n = 431) (Additional file 1: Figure S4). In addition, the analysis of the distribution of both scores in the CIT cohort according to the stages I to IV of CRC showed that the lowest levels were found in stage IV (Fig. 5c). In line with the above results, we found that the complex I score was highly prognostic for overall survival (OS) and relapse-free survival (RFS) in the CIT cohort, since patients with a low complex I score were associated with a worse outcome in terms of OS and RFS (Fig. 5d). Finally, gene set enrichment analyses (GSEA) were used to get insight into pathways most correlated to the expression of CI genes in the CIT cohort (Fig. 6). We selected the same three subunits representative of the CI catalytic core that we had studied at the protein level in our cell-based experiments, namely NDUFV1, NDUFS1 and NDUFS3, and found that the corresponding transcripts were inversely correlated to EMT pathway, which align well with the EMT signature of CMS4 tumors. We extended the analyses to an ex vivo transcriptomic dataset on a panel of CRC cell lines [15], according to the CMS classification that has been ascribed in the study by Sveen et al. [19]. The results showed in Additional file 1: Figure S5 corroborated the above observation that the CI score and Catalytic core score were downregulated in CMS4 cell lines (Additional file 1: Figure S5a) and that the expression of the catalytic core subunits NDUFV1, NDUFS1 and NDUFS3 were negatively correlated to EMT pathway (Additional file 1: Figure S5b).

## Discussion

In this paper we sought to characterize mitochondrial CI in colon cancer given the dual importance of this complex in mitochondrial energy metabolism and ROS generation, and by taking into account the heterogeneity of CRC.

As a starting point, we have exemplified that cellular models that recapitulate the features of CMS1 CRC – LoVo cells – or CMS4 CRC – MDST8 cells – exhibit significant differences in CI activity, which warrants taking into account the CMS classification in investigations of CRC mitochondrial metabolism. We further observed that the reduced CI activity monitored in MDST8 cells is accompanied and may likely be accounted for by reduced protein levels of several key subunits of the CI catalytic core. Despite lower CI activity in MDST8 cells, the “seahorse” data revealed similar mitochondrial ATP production capacities as compared with LoVo cells. These results led us to consider the possibility that the reduced CI activity in MDST8 cells may have a different purpose than reducing cellular ATP production and may endow cells with a non-energetic advantage. Because superoxide production has been shown to be inversely correlated to CI enzyme activity in inherited CI deficiency [20, 21, 29], we suspected an increased ROS production in MDST8 and, indeed, our results support this hypothesis, since we measured a heightened mitochondrial ROS production in MDST8 in coherence with the decreased CI enzyme activity.

It is now widely admitted that cancer cells exhibit elevated levels of cellular ROS compared to normal cells and that the ability of cancer cells to adapt to oxidative stress is key to different aspects of cancer development: initiation, promotion, progression, and metastasis [11]. While these observations have prompted the development of antioxidant strategies to suppress cancer development [30], clinical trials have failed to demonstrate a beneficial impact of dietary antioxidant supplementation [31]. In pre-clinical models, conflicting results have been reported as to whether buffering excessive ROS or instead further boosting ROS should be employed to interfere with cell migration [11, 30]. Importantly, in this field of research, the contribution of mtROS to the various stages of tumorigenesis remains understudied, and, how mtROS are generated and how they could drive EMT are in their infancy.

**Fig. 7:**
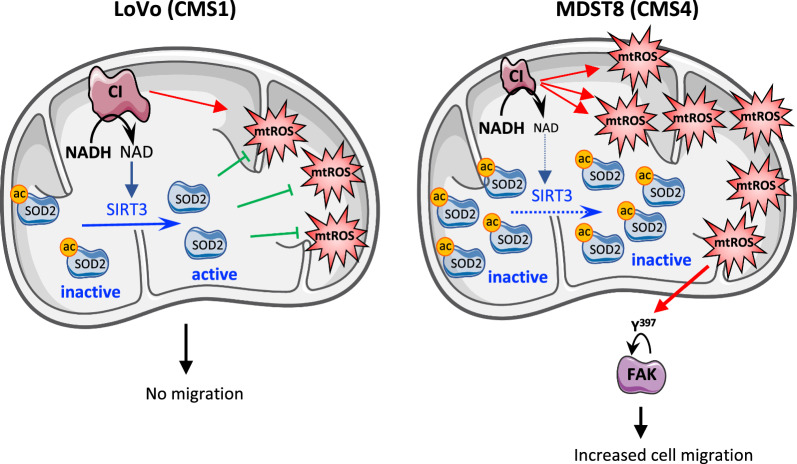
Hypothesis by which CI downregulation might foster MDST8 cell migration. The decrease in CI measured in MDST8 generates mtROS and hampers the production of NAD^+^, which become limiting for NAD^+^-dependent SIRT3 activity. Since, SIRT3 activity is mandatory for the induction of SOD2 activity by deacetylation, this results in decreased SOD2 enzyme activity in MDST8, contributing to maintain the levels of mtROS. In turn, these mtROS likely induce FAK activation, which participates to MDST8 migratory potential

Currently, the prevalent model is that the increased levels of cellular ROS, due to excessive metabolic activity, can be counteracted by an increase in their antioxidant capacities to allow the pro-tumorigenic effects of ROS, while avoiding their harmful effects. In this study, we showed that instead, the increase of mtROS production is accompanied by a decrease of SOD2 enzyme activity. In most studies, this assumption of the elevation in antioxidant defenses mainly relies on increases of mRNAs of transcription factors controlling key proteins, or proteins themselves, or increases in protein levels measured by western-blots or immunohistochemistry in tumors. For instance, in CRC, immunochemistry analysis has revealed a higher expression of SOD2 protein in tumor tissue than in the normal tissue adjacent [32]. Here, we show that although the SOD2 protein level is higher in MDST8 cells compared to LoVo, the SOD2 enzyme activity is lower due to its acetylated state. This result highlights the importance of i) assessing the biological function of any alterations found in cancer cells and ii) considering post-translational modifications of proteins as key points of regulation. This could contribute, at least in part, to explain the contradictory results found concerning the dichotomous role of SOD2 in cancer, to which a tumor suppression or a tumor promotion function has been attributed [22]. SOD2 is a protein finely regulated, at transcriptional, post-transcriptional and post-translational levels [22]. Thus, one post-translational modification of SOD2 is particularly relevant in the context of our study, namely the activation of SOD2 via deacetylation by SIRT3, a mitochondrial NAD^+^-dependent deacetylase, which belongs to the Sirtuins family [33, 34]. Indeed, the following cascade of events could explain why SOD2 enzyme activity is downregulated despite an increased level of SOD2 protein: the dysfunction of CI highlighted in this study likely limits the NAD^+^ availability in the cells, which in turn hampers the activity of SIRT3 leading to the acetylation/inactivation of SOD2. Of note, our group has already studied in depth and validated this scenario in primary fibroblasts of patients with recessively inherited isolated CI deficiency [20]. Moreover, decreased SIRT3 activity and acetylation of SOD2 have been reported in various cancers such as breast cancer and hepatocellular carcinoma [22]. Interestingly, the dichotomous role of SIRT3 in cancer, which has emerged recently [35], might be explained by its dependency to mitochondrial NAD^+^ pool and thus to mitochondrial NAD^+^ “supplier” like the mitochondrial CI. As already mentioned, the prevailing theory concerning redox homeostasis in cancer cells is that the cells respond to an elevated ROS production by increasing their antioxidant capacity. The results obtained in this study allow to propose an alternative and provocative model of a strategy developed by CRC cells (Fig. 7). Since mitochondria are the primary source of ROS, cancer cells slightly downregulate the CI activity to generate a graduate and moderate mtROS production that likely sustains the malignant phenotype. The CI dysfunction has also for consequence to limit the mitochondrial pool of NAD^+^, restraining the activity of pivotal NAD^+^-dependent proteins like SIRT3, which leads to SOD2 inactivation. Therefore, MDST8 cells, by downregulating their mitochondrial CI develop a “smart” cellular strategy, which allows an increase in mtROS while preventing SOD2 from functioning properly.

Thus, if one hypothesizes that MDST8 finely tune their CI activity to produce an optimal level of mtROS to sustain their migration, then it can be anticipated that manipulating ROS levels could shift cells in a less “favorable” state. Our results align well with this paradigm since we found that both rotenone, which boosts ROS levels above the cellular tolerability threshold, and MitoTempo, which buffers mitochondrial ROS, exerted a comparable negative impact on cell migration. These experiments indeed support the notion that MDST8 reside on a ridge line to ensure maximal migratory potential. Our hypothesis that MDST8 cells exquisitely adjust a favorable mtROS production to ensure maximal migratory potential is consistent with the results of Porporato et al. obtained in SiHa cells [36]. Indeed, these authors showed that a partial CI inhibition led to increase mtROS production that promoted SiHa tumor cell migration, and that, conversely MitoTempo, which lowered mtROS levels, decreased SiHa cell migration. Concerning the specific scavenging of mtROS with MitoTempo, our results are in line with those of Porporato et al. since we showed that MitoTempo slowed MDST8 cell migration. However, our results slightly differ since in their study, the moderate CI inhibition in SiHa cells was obtained using Rotenone while in our, the CI inhibition is already present in MDST8.

Our results also point to cell specific effects, since LoVo cells failed to migrate whether in basal conditions or after treatment, be it anti or pro-oxidant. Indeed, although LoVo cells exposed to rotenone produce ROS levels to a level comparable to that monitored in MDST8 cells at basal levels, no migration was induced, demonstrating that ROS elevation per se is not sufficient to promote cell migration. Instead, it is likely that the expression of EMT markers is a pre-requisite for ROS to exert a pro-migratory effect, as rotenone did not induce the expression of ZEB1 or N-cadherin (mRNA and protein) in LoVo cells (data not shown). However, and even though the anti or pro-oxidant mtROS manipulations did not act on LoVo migratory potential, it is noteworthy that both treatments, impacted LoVo behavior, since Rotenone and MitoTempo decreased cell’s proliferation.

Next, we wondered what mechanism could account for the link between mtROS elevation and migration in CRC cells. Several reasons prompted us to investigate the possible involvement of FAK in the transduction of promigratory effects of mtROS in MDST8 cells. Adhesion of cells to extracellular matrix is mandatory for their migration and is mediated by continuous formation and turnover of specific cellular structures called focal adhesions (FAs) [24]. FAK is known to be one of the main proteins involved in disassembly of FAs. Moreover, FAK is frequently overexpressed in different types of cancer [37], in which it plays an important role in malignant features such as EMT, and autophosphorylation of FAK is elevated in highly mobile and invasive malignant cells [24]. However, although during the last years, expression of total and activated FAK have been characterized in various cancers, including CRC [37, 38], the possible role of mtROS on their expression or on their subcellular localization needs further investigations.

Here, we found a strong staining of FAK and p-FAK-Y^397^ in MDST8 in coherence with their migratory phenotype. Indeed, since it has been shown that oxidative stress can inhibit phosphatases [39], it can be surmised that mtROS levels measured in MDST8 can (i) lead to increase p-FAK-Y^397^ level by inhibiting FAK dephosphorylation by phosphatase and (ii) prevent FAK degradation since it has been shown that dephosphorylation of FAK may be needed for its turnover [40]. In full agreement with the above hypothesis, we found a decrease in both p-FAK-Y^397^ and FAK steady state levels in MDST8 cells exposed to MitoTempo, which causes a reduced migration. The link between mtROS and the MDST8 migratory phenotype is further supported by the p-FAK-Y^397^ nuclear localization in MDST8, since recent data have shown that FAK could translocate into the nucleus in cells submitted to oxidative stress, and that this was associated with loss of cells’ attachment [26, 28]. Of note, our results again are consistent with those of Porporato et al., who showed that manipulating mtROS levels with rotenone or MitoTempo acts on tumor migration in SiHa cells through actions on FAK [36]. Besides, the reorganization of the MDST8’s cytoskeleton in the presence of optimal mtROS levels and its involvement in morphological changes required for cells’ migration, is attested by the formation of actin stress fibers [41]. Collectively, our observations provide evidence for a more active FAK in response to elevated mtROS, evidenced in MDST8 cells, likely explaining their migratory potential (Fig. 7).

Finally, the *in-silico* analysis of CRC patients’ datasets indicates that our ex vivo data have clinical relevance. Indeed, the transcriptomic analysis of the CIT cohort shows a decrease in both CI and catalytic core scores in all CMS groups compared to non-tumor samples, with highest level in CMS1 and lowest in CMS4. These results are reminiscent of the decrease of CI enzyme activity in MDST8 cells compared to LoVo cells, which exhibit specific features of CMS1 and CMS4 groups, respectively. Since, it is well acknowledged that colorectal tumors exhibit increased levels of different markers of oxidative stress compared to non-tumoral tissues [42], it can be surmised that the level of mtROS might be significantly increased in all CMS subtypes, compared to NT samples and might be the highest in CMS4 among tumors, due to an inversely correlated CI enzyme activity. These hypotheses are also supported by the robust anti-correlation between CI subunits and EMT pathway in CIT cohort. It is then tempting to speculate that reduced CI and increased mtROS account, at least partly, for the specific EMT signature of CMS4 tumors.

The purpose of this study was to investigate the mitochondrial CI taking into account the CMS classification of CRC in pre-clinical ex vivo models. Altogether, our data suggests that (i) MDST8 cells finely adjust a favorable mtROS level by regulating both mtROS production (downregulation of CI activity) and mtROS scavenging (decreased SOD2 activity) to ensure optimal migration potential (ii) increasing mtROS levels (Rotenone) above a certain threshold can have a similar effect on proliferation in LoVo (CMS1) and MDST8 (CMS4) but a different effect on migration, demonstrating that ROS elevation per se is not sufficient to promote migration (iii) scavenging mtROS (MitoTempo) has a different effect on proliferation in LoVo and MDST8, but also decreases MDST8 migration like rotenone (iv) Thus, in MDST8 cells, both increasing or decreasing mtROS could slow migration but likely through different mechanisms.

However, there are some limitations to our study. First, we do not provide direct evidence that downregulation of CI affects CRC cell migration in vivo*,* which would necessitate xenograft experiments with MDST8 cells. Second, we did not investigate the mechanism(s) by which CI is decreased in CRC cells, which would rely on in-depth-study of the possible molecular/cellular pathways leading to transcriptional downregulation. Conversely, several strengths of our work are worth mentioning. First, unlike many studies, we have characterized the biological consequences of alterations of proteins at a biochemical and functional level, such as for SOD2 protein and activity. Second, our data may contribute to reconcile apparently discordant findings of the literature regarding (i) the pro- and anti-migratory properties of ROS in cancer cells or (ii) the tumor-promoting or tumor-suppressive effect of SOD2. Last but not least, our findings highlight for the first time the necessity to take into account the CMS classification when examining metabolic features of CRC, and thereby extend the relevance of this classification to cell metabolism, beyond the “metabolic” label of the CMS3 subtype based on enrichment of metabolic pathways assessed through GSEA [3].

Concerning this last point, future studies should aim at re-centering the mitochondria in the overall landscape of CRC metabolism, with a particular attention to other mitochondrial respiratory chain complexes, and at investigating many other functions of these organelles beyond their role as energy provider. Future studies are also needed to further investigate the role of the nuclear localization of FAK in MDST8 cells.

In conclusion, we believe that our findings have multiple important basic and clinical implications and that taking into account the CMS-associated metabolic heterogeneity of CRC may provide new avenues for the development of diagnostic tools and therapeutic strategies.

## Supplementary Information

**Additional file 1: Figure S1.** LoVo and MDST8 cell lines are prototypical CMS1 and CMS4 models. (a) Total cell number at 48 h and 72 h. Cells were counted using the CASY TT cell counter. (b and c) Representative kinetics of LoVo and MDST8 proliferation/adhesion using xCELLigence methodology and quantification of cell index at 24 h (n = 8). (d and e) Representative kinetics of cell migration using xCELLigence and quantification of cell index at 12 h (n = 6). (f) Representative immunoblot and quantification of N-cadherin, Vimentin and ZEB1 protein levels by western-blot (n = 5). The results are means ± SEM of n independent experiments. In certain experiment, samples were run in triplicates or quadruplates. The p values are indicated in the figure. **Figure S2.** Rotenone diminishes proliferation of LoVo and MDST8 cells. (a and b) Dose response of rotenone on cell number in LoVo and MDST8 after 72 h of treatment. (n = 2, in each experiment, the determinations were performed in triplicates). **Figure S3.** The 7 subunits of the CI catalytic core are significantly decreased in CMS4. Relative expression of NDUFV1, NDSUV2, NDUFS1, NDUFS2, NDUFS3, NDUFS7 and NDUFS8 genes in patients from the CIT cohort (n = 566) according to the CMS classification. NT: non-tumors controls. **Figure S4.** Complex I scores are decreased in CMS4 subtype in the validation cohort. (a) Distribution of the Complex I score (mean of the 38 nuclear-encoded genes’ expression) and the Cat Core score (mean of the expression of the 7 subunits constituting the catalytic core of CI) in the validation cohort (n = 431) according to CMS classification. (b) Relapse-free survival according to high and low complex I score was determined in the validation cohort. Hazard ratios were adjusted for TNM stage, MMR status and adjuvant chemotherapy. **Figure S5.** Complex I scores are decreased in CMS4 subtype in CRC cell lines. (a) Distribution of Complex I score and Catalytic core score in a panel of CRC cell lines (n = 148) to which a CMS classification has been ascribed. (b) GSEA analysis showing that the expression of the catalytic core subunits NDUFV1, NDUFS1 and NDUFS3 is negatively correlated to EMT signature in the panel of CRC cell lines. NES: normalized enrichment score.

## Data Availability

All data generated or analyzed during this study are included in this published article [and its supplementary information files].
