# Supplementary figures and images for: Downregulation of mitochondrial complex I induces ROS production in colorectal cancer subtypes that differently controls migration
Source: J Transl Med. 2023 Aug 3;21:522. doi: 10.1186/s12967-023-04341-x (PMC10398918; doi:10.1186/s12967-023-04341-x)

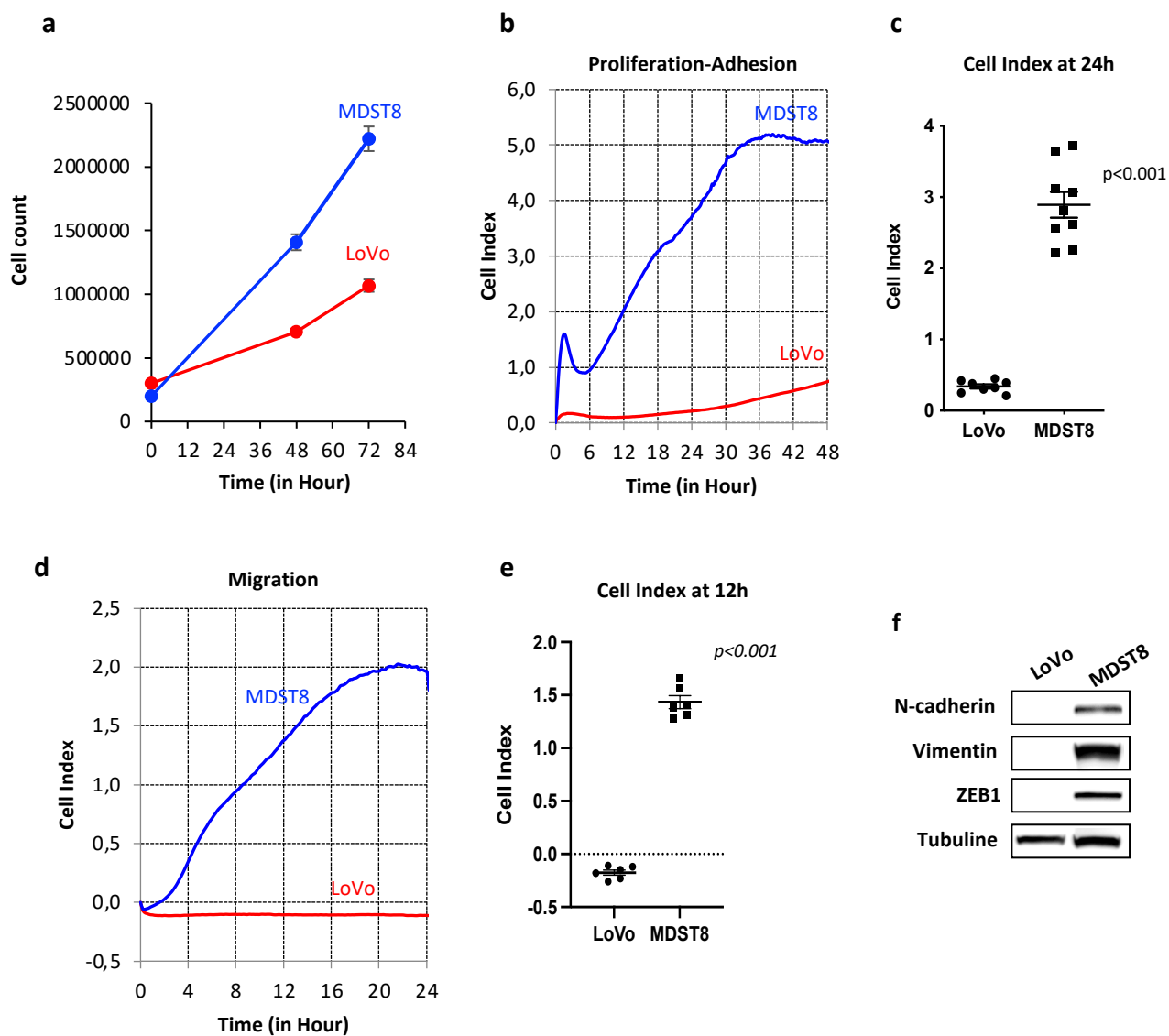

Supplemental Figure 1

**a**

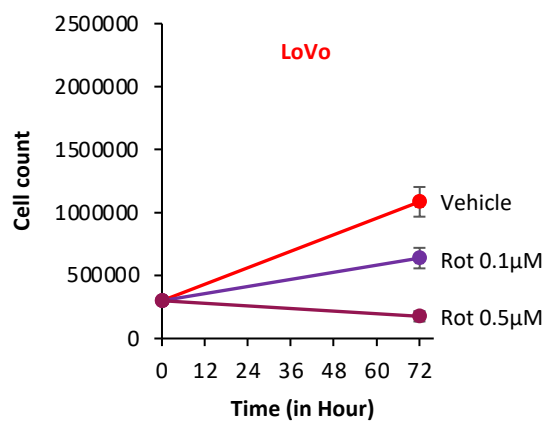

**b**

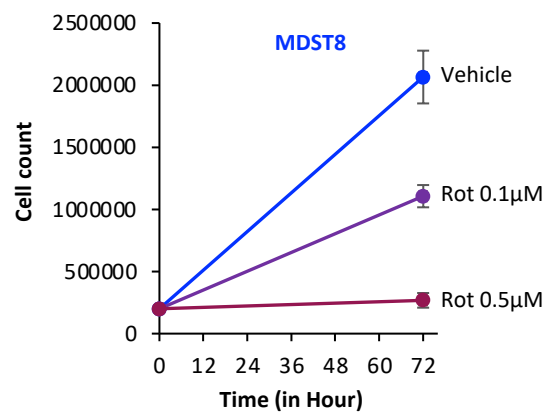

**Supplemental Figure 2**

**a**

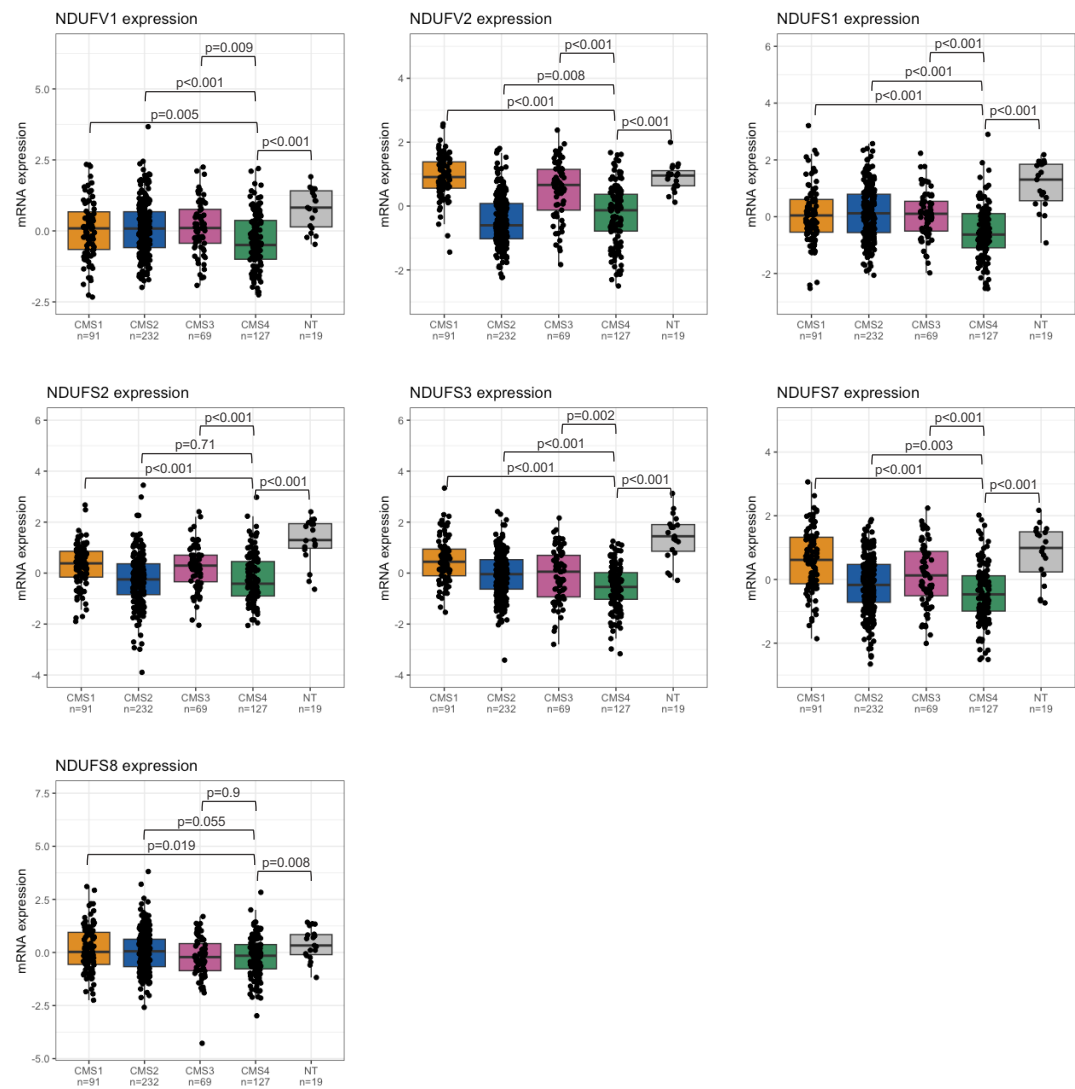

**Supplemental Figure 3**

a

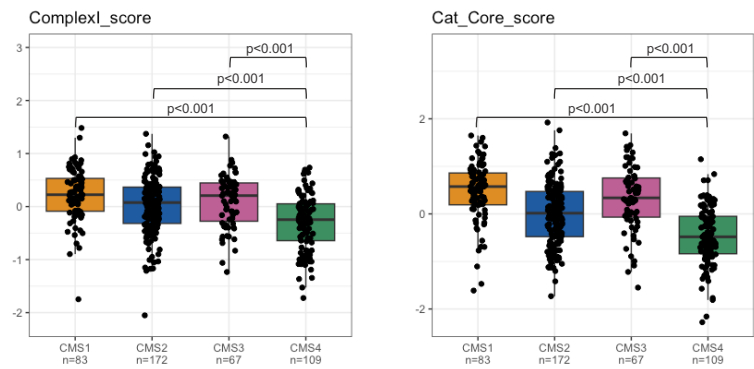

b

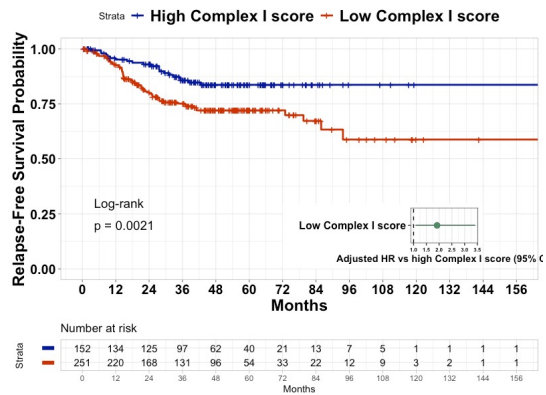

Supplemental Figure 4

**a**

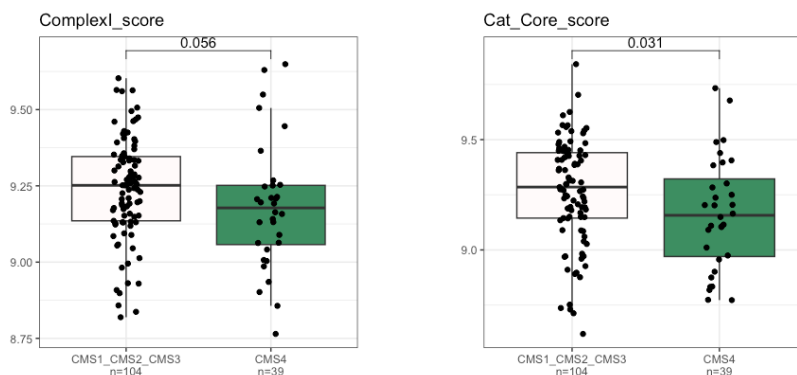

**b**

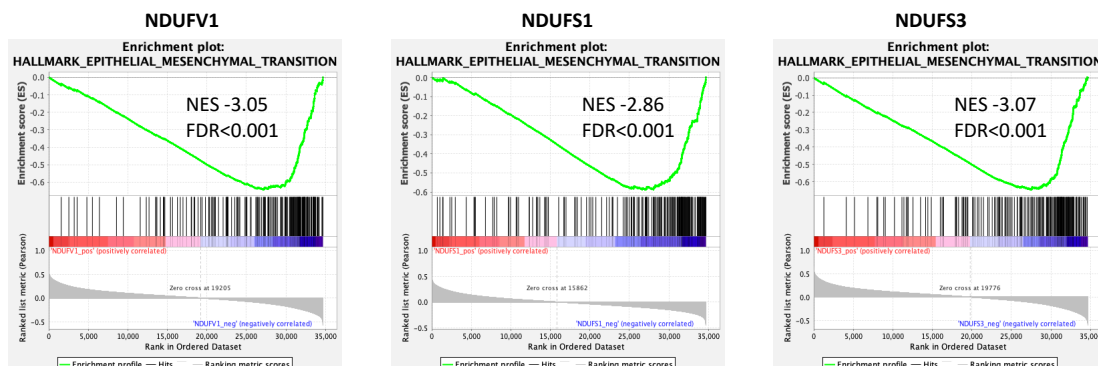

Supplement: Supplementary file 1 — Additional file 1: Figure S1. LoVo and MDST8 cell lines are prototypical CMS1 and CMS4 models. (a) Total cell number at 48 h and 72 h. Cells were counted using the CASY TT cell counter. (b and c) Representative kinetics of LoVo and MDST8 proliferation/adhesion using xCELLigence methodology and quantification of cell index at 24 h (n = 8). (d and e) Representative kinetics of cell migration using xCELLigence and quantification of cell index at 12 h (n = 6). (f) Representative immunoblot and quantification of N-cadherin, Vimentin and ZEB1 protein levels by western-blot (n = 5). The results are means ± SEM of n independent experiments. In certain experiment, samples were run in triplicates or quadruplates. The p values are indicated in the figure. Figure S2. Rotenone diminishes proliferation of LoVo and MDST8 cells. (a and b) Dose response of rotenone on cell number in LoVo and MDST8 after 72 h of treatment. (n = 2, in each experiment, the determinations were performed in triplicates). Figure S3. The 7 subunits of the CI catalytic core are significantly decreased in CMS4. Relative expression of NDUFV1, NDSUV2, NDUFS1, NDUFS2, NDUFS3, NDUFS7 and NDUFS8 genes in patients from the CIT cohort (n = 566) according to the CMS classification. NT: non-tumors controls. Figure S4. Complex I scores are decreased in CMS4 subtype in the validation cohort. (a) Distribution of the Complex I score (mean of the 38 nuclear-encoded genes’ expression) and the Cat Core score (mean of the expression of the 7 subunits constituting the catalytic core of CI) in the validation cohort (n = 431) according to CMS classification. (b) Relapse-free survival according to high and low complex I score was determined in the validation cohort. Hazard ratios were adjusted for TNM stage, MMR status and adjuvant chemotherapy. Figure S5. Complex I scores are decreased in CMS4 subtype in CRC cell lines. (a) Distribution of Complex I score and Catalytic core score in a panel of CRC cell lines (n = 148) to which [file 12967_2023_4341_MOESM1_ESM.pdf]
